# Recent developments in peptide vaccines against Glioblastoma, a review and update

**DOI:** 10.1186/s13041-025-01221-x

**Published:** 2025-06-13

**Authors:** Reza Salahlou, Safar Farajnia, Effat Alizadeh, Siavoush Dastmalchi

**Affiliations:** 1 Department of Medical Biotechnology, Faculty of Advanced Medical Sciences, Tabriz University of Medical Sciences, Tabriz, Iran; 2 Biotechnology Research Center, Tabriz University of Medical Sciences, Tabriz, Iran; 3 Drug Apploed Research Center, Tabriz University of Medical Sciences, Tabriz, Iran; 4 Student Research cmmittee, Tabriz University of Medical Sciences, Tabriz, Iran; 5 Department of Medicinal Chemistry, School of Pharmacy, Tabriz University of Medical Sciences, Tabriz, Iran; 6 Department of Medicinal Chemistry, School of Pharmacy Faculty of Pharmacy, Near East University, , P.O. Box 99138, Nicosia, Turkey

**Keywords:** Glioblastoma, Peptide vaccines, Immunity

## Abstract

Glioblastoma multiforme (GBM) is the most prevalent invasive CNS tumor, with a high incidence rate and a high likelihood of recurrence in most patients. Despite available treatments, recurrent glioblastoma (rGBM) exhibits growing resistance to chemotherapy and radiotherapy, which necessitates the development of newer methods of treatment. Peptide vaccines, a type of cancer immunotherapy, have recently attracted attention as a potentially practical therapeutic approach because they target tumor-associated or tumor-specific antigens to generate an effective immune response against cancer cells. These vaccines have been included in several clinical trials, demonstrating their safety and effectiveness by eliciting protective immune responses. However, peptide vaccines for glioblastoma face challenges due to the complex nature of intracranial brain tumors that require innovative approaches and in-depth research to increase their efficacy. The main topics covered in this article include immunological inhibitors and immune characteristics of the CNS and GBM, the basis of immunity, and the significant results of clinical trials of peptide vaccine therapy for GBM. Additionally, it examines the potential causes of the low effectiveness of these vaccines and recommends future research to address the specific challenges associated with immunotherapy in GBM. The evaluation of preliminary phase studies and phase III clinical trials will provide insights into potential immunological responses, biosecurity precautions, and clinical outcomes, guiding future vaccination initiatives to promote higher effectiveness.

## Introduction

Glioblastoma multiforme (GBM), a WHO grade IV glioma, is the most malignant kind of primary brain tumor. The rapid growth and invasive nature of this cancer make it a challenging cancer to treat. The average survival time for people with GBM is usually between 12 and 15 months. At 2-year and 5-year intervals, survival rates are relatively low, at about 10% and 25%, respectively [1].

Standard treatment for GBM involves the safest surgical resection of the tumor, followed by radiation therapy and temozolomide (TMZ) chemotherapy to maximize tumor removal, target residual cancer cells, and increase treatment efficacy [2].

Regretfully, because of the highly aggressive and diverse characteristics of GBM, the disease is always regressive, and none of the available treatments have shown significant efficacy in long-term survival after disease recurrence [3]. Recent advancements in immunotherapy research have shown promising results in treating various types of cancer. Immunotherapy activates the body’s immune system through immune-stimulating agents, oncolytic viruses, monoclonal antibodies, peptide vaccines, cellular immunotherapy, and immune checkpoint inhibitors, which aim to suppress tumor cell growth and trigger tumor cell apoptosis, resulting in beneficial therapeutic outcomes. The treatment of gliomas with immunotherapy faces numerous challenges due to the unique characteristics of central nervous system tumors located within the intracranial region, even with the advances made in immunotherapy through clinical investigations. These challenges include the difficulty of gaining access to the tumor site and the complications about the blood-brain barrier, which impede the efficacy of immunotherapeutic agents [4]. Peptide vaccines show high specificity due to the integration of peptide sequences derived from tumor-associated antigens (TSAs) or tumor-specific antigen targets (TAAs), which facilitates the direct production of antigens. However, their immunogenicity is lower, which may limit their ability to elicit strong resistance responses. TAA/TSA peptide-based vaccines face challenges in effectively presenting antigens and eliciting robust CD8^+^ T cell responses. This necessitates improvements in vaccine design to enhance clinical outcomes. By downregulating MHC class I expression or interfering with antigen processing pathways, such as by lacking TAP or losing beta-2 microglobulin (B2M), which impairs peptide presentation and decreases visibility to CD8^+^ T cells, tumor cells avoid immune detection [5]. Additionally, TAAs, as self-antigens, are limited by immune tolerance, leading to weak CD8^+^ T cell activation due to low-affinity TCRs or thymic deletion of high-affinity T cells [6, 7]. While tumor cells’ impaired proteasomes or TAP further restrict peptide availability, short peptides may bind MHC class I on non-professional antigen-presenting cells lacking costimulatory signals, promoting tolerance rather than immunity [8]. The tumor microenvironment, characterized by immunosuppressive elements such as TGF-β and regulatory T cells, along with the upregulation of PD-1, leads to T cell exhaustion, significantly hindering the functionality of CD8^+^ T cells [9, 10]. Moreover, MHC class I polymorphism restricts TAA peptide binding to specific HLA types, thereby reducing vaccine applicability across diverse patient populations [11]. Finally, short peptides often fail to engage CD4^+^ T cell help or costimulatory signals, resulting in transient CD8^+^ T cell responses without durable memory. Innovative strategies, such as synthetic long peptides, heteroclitic peptides, polyepitope constructs, personalized vaccines, adjuvants like CpG, and checkpoint inhibitors, are needed to enhance MHC class I presentation and CD8^+^ T cell responses, improving clinical outcomes [6, 7, 9, 12]. This article examines the immunological foundation and significant clinical trial findings of peptide vaccine treatment for GBM, assesses the reasons for its lack of effectiveness, and proposes strategies to overcome the particular obstacles associated with immunotherapy for GBM using peptide vaccines. An accurate understanding and complete description of how peptide vaccines are used and the barriers facing GBM can be important in advancing new treatment strategies in clinical studies and future basic research. This insight will pave the way for improved treatment methods and scientific advances in GBM.

### Immune microenvironment of GBM

**Fig. 1:**
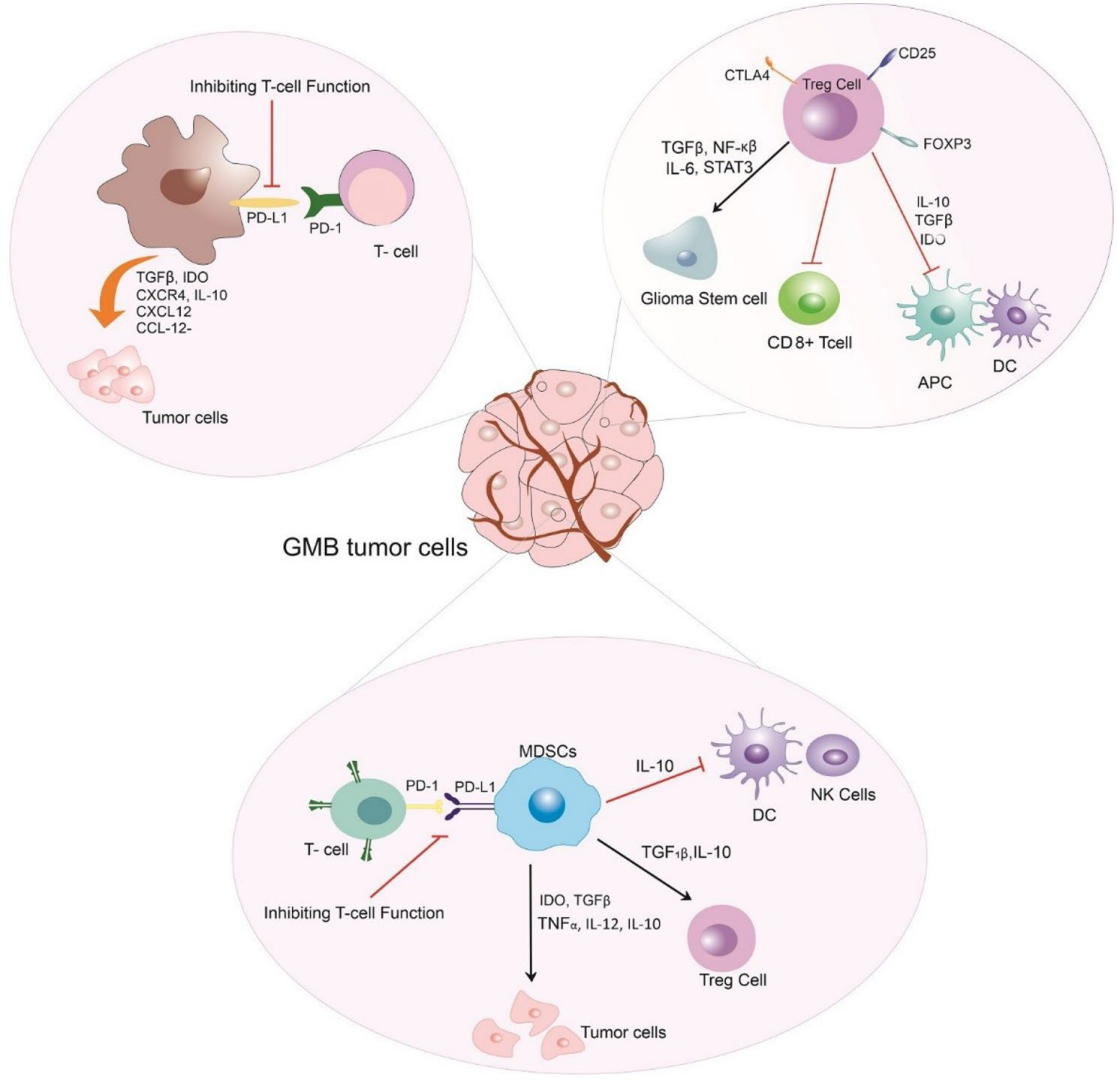
Immunosuppressive microenvironment of glioblastoma

The immune microenvironment of GBM is a complex and dynamic landscape that significantly influences the efficacy of immunotherapeutic strategies. GBM creates an immunosuppressive microenvironment that allows it to evade the body’s natural immune responses, posing a formidable challenge for treatment (Fig. 1).

Key Components of the GBM Immune Microenvironment:

### Myeloid-derived suppressor cells (MDSCs)

MDSCs, characterized by the expression of CD11b^+^, CD33^+^, and low levels of HLA-DR, represent a population of immature myeloid cells exhibiting considerable diversity and are pivotal in mediating immunosuppression induced by tumor cells [13]. These cells play a crucial role in creating an immunosuppressive environment by suppressing the activation and proliferation of T cells. MDSCs that block innate anti-tumor immunity are more prevalent in the bloodstream of GBM patients. MDSCs’ role is sex-dependent, with monocytic MDSCs (M-MDSCs) promoting GBM progression in males and granulocytic MDSCs (G-MDSCs) regulating immune responses [14]. Human M-MDSCs are typically identified as CD11b^+^CD14^+^HLA-DR^−^/lowCD15^−^CD33^high^ cells [15]. These cells, derived from monocytic precursors, are part of the mononuclear phagocyte system and can differentiate into macrophages or dendritic cells (DCs) under certain conditions. Through various processes, including the synthesis of arginase-1, nitric oxide, inducible nitric oxide synthase (iNOS), as well as the production of immunosuppressive cytokines such as IL-10 and TGF-β, M-MDSCs inhibit T-cell proliferation and function. These elements encourage immune evasion by suppressing CD8^+^ T-cell activity and natural killer (NK) cell cytotoxicity [16]. M-MDSCs help grow GBM tumors by encouraging cancer stem cell (CSC) and epithelial-mesenchymal transition (EMT) phenotypes and aiding in tumor cell dissemination. They generate chemokines and growth factors, such as CCL3, CCL4, and CCL5, that attract regulatory T cells (Tregs) to the TME, thereby intensifying immunosuppression [17, 18]. G-MDSCs are often described as HLA-DR^−^CD33^Mid^CD11b^+^CD15^+^CD14^−^ cells [15]. These are derived from granulocytic lineage and resemble neutrophils. Reactive oxygen species (ROS), arginase-1, and G-CSF are the main ways that G-MDSCs inhibit immune responses in an antigen-specific way. These elements aid immune evasion by suppressing T-cell proliferation and NK cell activity. In GBM, G-MDSCs are more prevalent in tumor tissue than in peripheral blood, where they use arginase and S100A8/9 to inhibit T-cell function [19, 20]. By secreting VEGF and reversing EMT/CSC phenotypes, G-MDSCs in GBM enhance cell proliferation in metastatic sites and promote tumor angiogenesis and metastatic growth [17]. Chemokine CCL2 is implicated in MDSC infiltration into the GBM microenvironment, enabling tumor-recruiting by CCR2^+^ cells. GBM expresses CCL2 and CCL7, which facilitate tumor recruitment by CCR2^+^ cells. Loss of CCR2 expression reduces MDSC outflow, reducing GBM infiltration. CCL2 mediates MDSC migration, inhibiting NK and T cell killing, Treg development, and DC maturation, thus inhibiting innate and adaptive immunity [20, 21]. Because MDSCs have a high glycolysis flux, they can exploit metabolic pathways to grow from bone marrow precursors. This mechanism indirectly inhibits effector T-cells by consuming carbon sources [22]. Large amounts of TNF-α, TGF-β, IL-12, IL-10, and IDO are released by MDSCs in an attempt to inhibit the immunological response to immunotherapy [23].

### Glioma-associated macrophages/microglia (GAMs)

They frequently take on an M2 phenotype, facilitating immunosuppression and tissue healing and supporting the formation of tumors. GAMs comprise around 30% of the tumor mass and comprise extra-parenchymal macrophages, BMDMs, and brain-resident microglia. The involvement of these cells relates to immunosuppression since they secrete IL-6, TGFβ1, PDGF, VEGF, periostin, MMP2, and MMP9. This encourages angiogenesis, the advancement of glioma tumors, their invasive behavior, and the development of distant pre-metastatic niches. In contrast, under certain circumstances, such as exposure to pro-inflammatory signals like IFN-γ, LPS, or GM-CSF, GAMs can polarize toward an M1-like phenotype. M1-like GAMs have anti-tumor effects by phagocytic activity, which involves invading tumor cells, and antigen-presenting functions, which trigger cytotoxic T cells. They also express markers like CD80, CD86, MHC-II, and iNOS. These cells enhance immune surveillance and tumor-killing by producing pro-inflammatory cytokines, such as TNF-α and IL-12 [24, 25]. Therapeutic attempts to target GAMs have included the use of CSF1R inhibitors, which aim to deplete or reprogram pro-tumorigenic macrophages. However, clinical trials have mostly failed to improve patient outcomes, as seen in the testing of the CSF1R inhibitor PLX3397 in GBM. The reasons for these failures include the depletion of both pro- and anti-tumor GAM populations, compensatory recruitment of immunosuppressive cells, and incomplete depletion of GAMs, which may interfere with advantageous immune responses. Further limiting efficacy are resistance mechanisms, such as the upregulation of alternative survival pathways in GAMs. These results emphasize the necessity of more accurate methods to preserve or improve M1-like functions while targeting M2-like GAMs selectively [26, 27]. Furthermore, GAMs induce an immunosuppressive microenvironment that inhibits T cell proliferation, increases T cell apoptosis, and inhibits cytotoxic T lymphocyte progression [28]. Research has demonstrated that GAMs secrete IL-10, CXCR4, IDO, TGF-β, CCL20, CXCL12, CCL22, and other substances, and they also substantially express PD-L1, which mediates and balances tumor immune activity [23].

### Regulatory T cells (Tregs)

Tregs are a subpopulation of CD4^+^ T lymphocytes with high expression of CD25, Foxp3, and CTLA-4, and Foxp3 regulates CTLA-4 expression in Tregs [14]. Tregs can inhibit robust anti-tumor immune responses and aid in maintaining immunological tolerance. Tregs stimulate the stemness of glioma cells via the TGF-β–NF-κB–IL6–STAT3 signaling pathway, which results in augmented tumor growth and aggressiveness [29]. Further research showed a relationship between the grade of glioma tumors and Treg infiltration. In contrast to grades III and II, grade IV tumors had the highest levels of FoxP3 expression. Similarly, grade IV tumors exhibited higher levels of heme oxygenase-1 (HO-1) mRNA expression in CD4^+^ CD25^+^ Tregs compared to grades III and II. The strong correlation between HO-1 expression and CD4^+^ CD25^+^ FoxP3^+^ Treg infiltration suggests that HO-1 plays a role in FoxP3-mediated immune suppression during the progression of gliomas [30–32]. Tregs primarily suppress DCs, APCs, and other lymphocytes by stimulating immunosuppressive molecules like IL-10, TGF-β, and IDO, thereby establishing an immunosuppressive microenvironment [33]. Furthermore, following α-PD-1 treatment, Tregs show increased lipid metabolism, localize within the tumor microenvironment and impede the responses of CD8^+^ T cells, reducing immune checkpoint inhibition effectiveness [34].

### Immune checkpoint inhibitors (ICIs)

Immune checkpoint inhibitors (ICIs) are monoclonal antibodies that target surface receptors to block pathways that inhibit T-cell activation, enhancing immunotherapy and restoring T-cell function. The domain of immune checkpoint suppression focuses on two key receptors: Cytotoxic T Lymphocyte-Associated Protein 4 (CTLA-4) and Programmed Cell Death Protein 1 (PD-1). These receptors are crucial in immunotherapy due to their established immune regulation functions and clinical success in treating various cancers, including GBM. Additional checkpoints, including Lymphocyte Activation Gene 3 (LAG-3), T-cell immunoglobulin and Mucin Domain-Containing Protein 3 (TIM-3), T-cell immunoreceptor with Ig and ITIM Domains (TIGIT), Indoleamine 2,3-Dioxygenase 1 (IDO1), and V-Domain Ig Suppressor of T Cell Activation (VISTA), are being explored for their potential in combination therapies, particularly in difficult-to-treat cancers like GBM [35, 36]. CTLA-4 and PD-1 are key immune checkpoint receptors that suppress T-cell activity, making them crucial targets for cancer immunotherapy. They act as inhibitors on T cells, which tumors use to evade detection, and blocking them with monoclonal antibodies enhances cancer immunity. Activated T cells and Tregs express CTLA-4, which inhibits early T-cell priming in lymphoid organs by competing with CD28 for B7 ligands on antigen-presenting cells. PD-1, expressed on activated T cells, B cells, and macrophages, binds to PD-L1/PD-L2 on tumor cells, inhibiting effector functions within the tumor microenvironment during the immune response. While PD-1 controls sustained T-cell responses in peripheral tissues, CTLA-4 controls early T-cell activation. Overall, PD-1 blockade demonstrates greater tumor specificity and results in fewer severe side effects, while CTLA-4 blockade induces more extensive immune activation and increased incidence of immune-related toxicities [37, 38].

The phase III clinical trial (NCT02617589) found that TMZ plus radiotherapy had a longer median overall survival (mOS) than with anti-PD-1 blockade (nivolumab) plus radiotherapy in individuals with GBM [39]. Therapies targeting CTLA-4, particularly the anti-CTLA-4 antibody (ipilimumab), are being actively investigated in multiple clinical trials for the treatment of GBM. A Phase I trial (NCT02311920) assessed the safety and dosing of ipilimumab, nivolumab, or their combination alongside TMZ in newly diagnosed glioblastoma patients, finding the regimen well-tolerated with no Grade 4 adverse events, achieving a mOS of 20.7 months and progression-free survival of 16.1 months [40].

Other trials, including NCT02829931, investigated the combination of ipilimumab and nivolumab with anti-VEGF (bevacizumab) and hypofractionated stereotactic re-irradiation for recurrent high-grade gliomas [41]. At the same time, NCT04606316 investigates the safety and efficacy of ipilimumab and nivolumab with surgery for rGBM [42]. The study examines the use of next-generation checkpoints like LAG-3, TIM-3, TIGIT, IDO1, and VISTA to overcome resistance to CTLA-4/PD-1 inhibitors, particularly in GBM, where the immunosuppressive tumor microenvironment presents significant challenges. It provides an overview of the mechanisms and their relationships to CTLA-4 and PD-1.

LAG-3 is expressed on activated B cells, T cells, NK cells, and DCs. It binds to MHC class II and galectin-3, which inhibits T-cell proliferation and cytokine production. Unlike CTLA-4, which acts early in T-cell priming, LAG-3 modulates sustained T-cell responses, often synergizing with PD-1 [36, 43]. Clinical trials, such as NCT02658981, investigate the combination of anti-LAG-3 (relatlimab) and nivolumab in rGBM, demonstrating modest survival advantages. The study revealed that the median survival of a group receiving latelimab treatment was 8.5 months, whereas a group receiving a combination of both treatments had a median survival of 8 months [44].

TIM-3 is expressed on mature T cells and macrophages, where it interacts with galectin-9 and CEACAM1, leading to T-cell exhaustion and immune suppression. In contrast to PD-1, it functions during the later phases of T-cell dysfunction, positioning it as a viable target for combination therapies [36, 45]. For patients with rGBM, a phase I trial (NCT03961971) is now examining the side effects of stereotactic radiosurgery combined with anti-TIM3 (MBG453) and anti-PD-1 (spartalizumab) [46].

TIGIT is an inhibitory receptor in T cells and NK cells that interacts with CD155/CD112, competing with co-stimulatory receptors such as CD226. Its function overlaps with PD-1 in inhibiting effector T-cell activity [47]. Trials like NCT04656535 evaluate anti-TIGIT (AB154) with anti-PD-1 (AB122) in rGBM [48].

IDO1 is an immunosuppressive enzyme that is overexpressed in GBM. It depletes tryptophan and produces kynurenine, which inhibits T-cell activity. In contrast to receptor-based checkpoints, IDO1 functions enzymatically, complementing PD-1 blockade [49]. A Phase I trial (NCT04047706) is presently studying the potential side effects of administering nivolumab, BMS-986,205 (an IDO1 inhibitor), and conventional radiation therapy, either alone or in combination with TMZ, to patients with newly diagnosed GBM [50].

VISTA is primarily expressed in microglia and macrophages within the central nervous system, inhibiting CD4^+^ T-cell activation and antigen-presenting cell activity. The role of this factor in GBM remains underexplored; however, it may contribute to the immunosuppressive tumor microenvironment and potentially enhance the effects of PD-1 or CTLA-4 blockade [51].

### Immunotherapeutic targets

Tumors express numerous TAAs and TSAs, which can be used as targets for immunotherapy. TSAs such as PTEN, TP53, H3.3 K27M, EGFRvIII, and IDH1 R132H are specific to tumor cells and arise from genomic mutations or errors in post-transcriptional/translational mechanisms [52]. TAAs are antigens found in tumor cells that are also expressed in normal cells; they can trigger different immune responses. TAAs include aberrantly expressed proteins, germline-restricted (cancer/testis), and differentiation-associated proteins. Numerous investigations have been conducted into the expression of TAAs in GBM, identifying several promising candidates for vaccine-directed immunotherapy. This has led to the discovery of dozens of TAAs associated with GBM, such as CTCFL, ACTL8, TERT, WT-1, OIP5, XAGE3, IL-13Rα2, EGFR vIII, IL-4, gp100, survivin, MAGE-1, CD133, TRP-2, AIM-2, HER2, EphA2, and YKL-40, which have been evaluated in early-stage therapeutic preclinical trials to show their safety and immunogenicity in the human body [53–55]. Validation is still required to demonstrate that there is a therapeutic window in which vaccines against the tumor can produce sufficient immunity to achieve clinical efficacy without causing severe systemic autoimmune symptoms, while retaining a low level of expression in normal tissues. Due to the possibility of TAA expression in healthy cells, TSAs may provide more suitable targets for vaccines than TAAs. In addition, TSAs are not affected by central immunological tolerance and have greater immunogenicity and affinity for MHC than TAAs [56]. Therefore, autoimmunity is a significant concern when targeting antigens on healthy cells because the immune system has a strong cytotoxic potential. Several studies have demonstrated that myelin-specific antibodies, such as those against myelin oligodendrocyte glycoprotein and proteolipid protein, can induce experimental autoimmune encephalomyelitis [57].

### Peptide vaccine

**Fig. 2:**
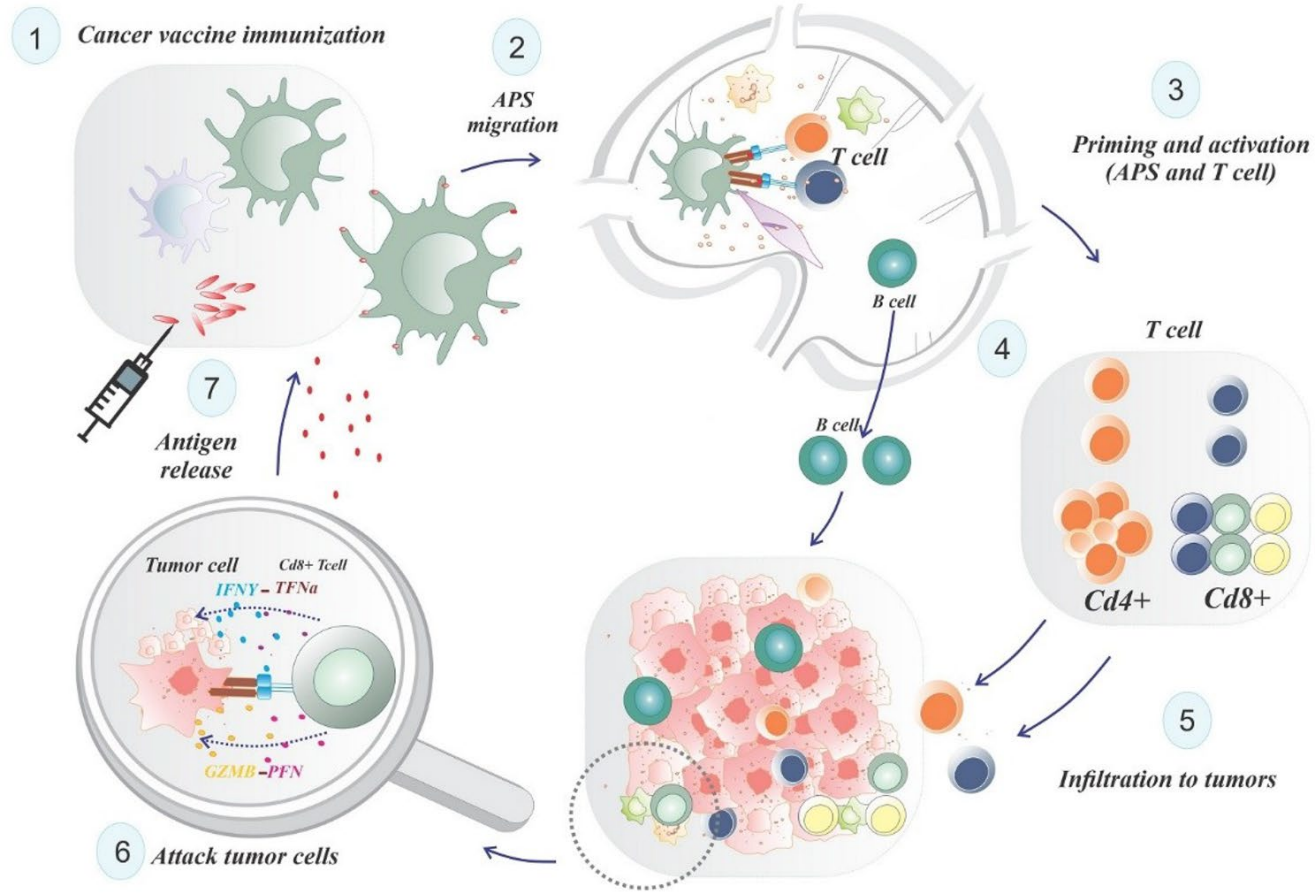
Principle of cancer peptide vaccines. Following the administration of the tumor vaccine, tumor-derived antigens are internalized and processed by DCs, which are then presented to major histocompatibility complex (MHC) class II or class I molecules through cross-presentation pathways. Antigen-presenting dendritic cells migrate to the lymphatic nodes, facilitating the recruitment and activation of various immune cell types. T cells that have been activated multiply and develop into effector and memory T cells. Effector T cells go to the tumor microenvironment (TME) and either directly destroy tumor cells or trigger their apoptosis. To boost the intensity and scope of the immune response in ensuing cycles, immunogenic dead tumor cells might produce TAAs and danger-signaling molecules

Peptide-based cancer vaccines utilize TSA/TAA-derived peptide sequences to elicit targeted immune responses with antineoplastic and immunomodulatory effects. These vaccines are categorized into short peptide epitopes (8–11 amino acids) and synthetic long peptides (SLPs, 25–30 amino acids) [6]. Short peptides bind directly to MHC class I molecules without requiring internalization, enabling rapid presentation to CD8^+^ T cells. However, their binding to MHC class I on non-antigen-presenting cells (non-APCs), which lack costimulatory molecules, may lead to suboptimal T-cell activation and immune tolerance [58]. In contrast, SLPs require processing by antigen-presenting cells (APCs), such as DCs, to present MHC class I-restricted epitopes via cross-presentation and MHC class II-restricted epitopes [59]. Following administration, APCs internalize SLPs, process them, and present epitopes to CD4^+^ and CD8^+^ T cells. CD8^+^ cytotoxic T lymphocytes (CTLs), activated by MHC class I-presented epitopes, target tumor cells by releasing perforin and granzymes or inducing apoptosis through Fas ligand interactions [60]. Concurrently, CD4^+^ T-helper (Th1) cells, activated by MHC class II-presented epitopes, secrete IFN-γ to enhance CTL activity and promote an inflammatory tumor microenvironment [61]. To boost immunogenicity, TAA-derived peptides are often combined with adjuvants, such as toll-like receptor ligands, which enhance DC maturation and antigen presentation [62]. Mature DCs migrate to lymph nodes, priming naïve T cells and driving differentiation into effector and memory T cells specific to TAAs. This process amplifies the cancer-immunity cycle, fostering sustained tumor-specific immunity [63] (Fig. 2). By targeting TAA-specific epitopes, peptide vaccines provide a precise and adaptable immunotherapeutic strategy, potentially expanding the antigenic repertoire and improving clinical outcomes. The widely recognized GBM vaccines include peptides like SurVax (survivin) and rindopepimut (EGFRvIII). A subsequent section will provide further information regarding the existing GBM peptide vaccines (Table 1).

**Table 1:** Current phase I/II/III clinical trials of GBM peptide vaccines

Target antigen	Antigen Classification	Experimental Treatment Arm	Phase	Status	NCT Identifier
EGFRvIII					
	TSA	Rindopepimut + GM-CSF + TMZ	Phase II	Completed	NCT00458601
	TSA	CDX-110 + GM-CSF + TMZ	Phase III	Completed	NCT01480479
	TSA	Rindopepimut + GM-CSF + Bevacizumab	Phase II	Completed	NCT01498328
	TSA + TAA	Live-attenuated, L. monocytogenes encoding EGFRvIII + NY-ESO-1	Phase I	Completed	NCT01967758
H3.3 K27M					
	TSA	H3.3-K27M peptide + Nivolumab + Poly-ICLC	Phase I- II	Completed	NCT0296023
	TSA	H3.3-K27M peptide + Poly-ICLC	Phase I	Recruiting	NCT04749641
	TSA	H3K27M peptide vaccine + Imiquimod + Tecentriq^®^	Phase I	Recruiting	NCT04808245
R132H					
	TSA	IDH1 peptide vaccine + TMZ + RT	Phase I	Completed	NCT02454634
	TSA	IDH1R132H peptide vaccine + Avelumab	Phase I	Active	NCT03893903
	TSA	PEPIDH1M vaccine + Td + TMZ	Phase I	Completed	NCT02193347
	TSA	PEPIDH1M vaccine + vorasidenib	Phase I	Not yet recruiting	NCT05609994
Survivin					
	TAA	Montanide ISA-51/Survivin Peptide Vaccine + Sargramostim	Phase I	Completed	NCT01250470
	TAA	SVN53-67/M57-KLH Peptide Vaccine + Temozolomide + Montanide ISA 51 VG + Sargramostim	Phase II	Active	NCT02455557
	TAA	SurVaxM	Phase II	Active	NCT05163080
	TAA	SurVaxM + Sargramostim + Montanide ISA 51 + Pembrolizumab	Phase II	Completed	NCT04013672
	TAA	SurVaxM + Montanide ISA 51 + sargramostim+	Phase I	Recruiting	NCT04978727
WT-1					
	TAA	modified 9-mer WT1 peptide + Montanide ISA51	Phase II	Completed	Not available
	TAA	modified 9-mer WT1 peptide (CYTWNQMNL) TMZ + RT	Phase I	Completed	Not available
	TAA	cocktail vaccine: WT1-peptide (WT1235, WT1332)	Phase I	Completed	Not available
	TAA	DSP-7888 Dosing Emulsion	Phase I	Completed	NCT02498665
	TAA	DSP-7888 Dosing Emulsion + Bevacizumab	Phase III	Completed	NCT03149003
	TAA	WT2725	Phase I	Completed	NCT01621542
TERT					
	TAA	UCPVax + Temozolomide	Phase II	Active	NCT04280848
Multipeptide					
	TAAs	SL-701; poly-ICLC + Bevacizumab + GM-CSF + Imiquimod	Phase I- II	Completed	NCT02078648
	TAAs	IMA950/Poly-ICLC	Phase I- II	Completed	NCT01920191
	TAAs	IMA950/Poly-ICLC and pembrolizumab	Phase I- II	Active	NCT03665545
	TAAs	EO2401((IL13Rα2,BIRC5,FOXM1,andUCP2) + nivolumab + bevacizumab	Phase I- II	Active	NCT04116658
	TAAs	P30-EPS (P30-linked EphA2, CMV pp65, and surviving)			NCT05283109
	TAAs	Multipeptide plus XS15)Survivin, Y-box binding protein 1, and others(+ temozolomide + RT	Phase I	Recruiting	NCT04842513
Personalized peptide					
	TSA	APVAC1/2 vaccine + Poly-ICLC + GM-CSF	Phase I	Completed	NCT02149225
	TSA	NeoVax + Pembrolizumab + RT	Phase I	Recruiting	NCT02287428
	TSA	NeoVax + Nivolumab + Ipilimumab	Phase I	Terminated	NCT03422094
	TSA	Peptides + Poly-ICLC + Tumor Treating Fields	Phase I	Active	NCT03223103

### **Epidermal** growth factor receptor variant type **III**

The EGFRvIII mutation, a type III epidermal growth factor receptor mutation found in 20–30% of GBM patients, results from the deletion of exons 2–7 in the EGFR gene. This deletion causes a truncation of the EGFR protein, allowing continuous activation [64]. The protein triggers a tyrosine kinase, initiating signaling via the RTK/RAS/PI3K pathway, leading to tumor development. Genetic rearrangement in the EGFRvIII protein leads to a distinct peptide sequence, affecting GBM patients’ survival. This highlights its importance as a target for anti-tumor immunotherapeutic strategies [65]. Rindopepimut is an immunotherapeutic vaccine with a 14 amino acid peptide synthesized from EGFR vIII, encapsulating the mutation locus and binding to keyhole limpet hemocyanin (KLH) for enhanced immune response [66]. The ACT III Phase II clinical trial (NCT00458601), a single-arm multicenter study, evaluated rindopepimut combined with TMZ in 65 patients with newly diagnosed GBM that expressed EGFRvIII. The trial achieved a mOS of 21.8 months and a 3-year survival rate of 26%, demonstrating extended survival compared to historical controls and highlighting rindopepimut’s potential efficacy [67]. Following this, the phase III ACT IV trial (NCT01480479), a randomized, placebo-controlled study, enrolled 745 patients with newly diagnosed GBM, comparing rindopepimut plus TMZ to a control group receiving KLH plus TMZ. The mOS was 20.1 months for the rindopepimut group and 20.0 months for the control group, showing no significant difference in survival [68]. Despite this, optimism persisted for rindopepimut’s use in rGBM. The ReACT phase II trial (NCT01498328) randomized 73 patients with rGBM into rindopepimut (*n* = 36) and control (*n* = 37) groups. Rindopepimut was well-tolerated, with transient, low-grade local reactions as the primary adverse effects. The 6-month progression-free survival (PFS6) rate, the primary endpoint, was 28% for rindopepimut versus 16% for the control group. Secondary and exploratory endpoints showed a significant survival advantage (HR = 0.53), higher objective response rate (30% vs. 18%), longer median response duration (7.8 vs. 5.6 months), and a 33% steroid-free rate at 6 months (vs. 0% in controls). Notably, 80% of rindopepimut-treated patients developed high anti-EGFRvIII antibody levels, linked to improved survival (HR = 0.17) [69]. Despite the small sample size, these results support rindopepimut’s potential in rGBM, which is pending further validation. Additionally, a Phase I trial (NCT01967758) investigated ADU-623, a bivalent vaccine using live-attenuated Listeria monocytogenes that expresses NY-ESO-1 and EGFRvIII antigens to enhance immune responses against high-grade astrocytic cancers. Safety and efficacy data for this trial are not yet available [70].

### H3.3 K27M

Diffuse midline gliomas (DMGs) primarily affect children and adolescents, arising in the central nervous system’s midline structures, such as the thalamus, brainstem, and spinal cord. These entities are characterized by a recurrent point mutation in the H3F3A gene, which codes for histone H3.3. Mutations in the HIST1H3B or HIST1H3C genes encoding histone H3.1 are also detected. A substitution of amino acid, specifically lysine, to methionine at position 27 (K27 M) induces a reduction in trimethylation of H3 K27, leading to demethylation at a global level and abnormal gene expression by inhibiting polycomb repressor complex 2 (PRC2). Diffuse intrinsic pontine gliomas (DIPGs) are the most common type of DMGs, comprising 10–20% of all brain tumors found in children [71, 72]. The primary treatment for H3 K27M mutant DMG is local radiation therapy, as TMZ and other chemotherapeutic agents are ineffective. With a median overall survival of less than a year and a two-year survival rate below 10%, H3.3 mutant tumors in DIPG have a poor prognosis because they are more aggressive and less radiation-responsive than H3.1 mutant or wild-type H3.3 tumors [73]. The H3.3 K27M mutation is consistently present across the whole tumor, similar to the IDH1 R132H mutation in adult gliomas, making it a suitable target for immunotherapy [74, 75]. Researchers have developed H3.3 10mer (p26–35) and 27mer (p14–40) peptide vaccines that bind to HLA-A*0201 and HLA-DR1, stimulating mutation-specific immune responses in preclinical models. They also created a CD8^+^ T cell clone using a synthetic peptide carrying the H3.3K27M mutation. These TCR-transduced HLA-A2^+^ T cells effectively eliminated HLA-A2^+^ H3.3K27M + glioma cells, inhibiting glioma growth in mice xenografts. Alanine-scanning assays confirmed the TCR’s safety for clinical use, providing a solid foundation for vaccines and TCR-T-cell treatments [76, 77]. The Pacific Pediatric Neuro-Oncology Consortium (PNOC) conducted the PNOC007 trial (NCT02960230) to evaluate the safety and effectiveness of an H3.3 K27 M-peptide vaccine in 19 newly diagnosed HLA-A*0201^+^ DIPG patients, administered with poly-ICLC post-standard treatment. This trial reported no grade-4 adverse effects, with overall survival rates of 40% for stratum A and 39% for stratum B and mOS of 16.1 months in patients with H3.3K27M-reactive CD8^+^ T cells, significantly longer than those without this response. Lower myeloid-derived suppressor cell levels were associated with extended OS, while dexamethasone reduced immune responses [78].

A study found that a patient treated with an H3K27M peptide vaccine achieved complete remission. The vaccine activates the immune system by expanding mutation-specific T cells and eliciting B cell responses. It has 34 unique T cell receptors and H3K27 M-reactive B cell receptors in activated B cells, demonstrating its ability to stimulate T and B cell responses for antibody production against tumors [79]. In a study by Grassel et al., eight adults with advanced H3K27M^+^ diffuse glioma received the H3K27M-specific long peptide vaccine (H3K27M-vac), with five also receiving anti-PD-1 therapy, resulting in safe, repeated vaccinations and mutation-specific CD4 + T cell responses in five patients across various HLA types, with a median PFS of 6.2 months, a mOS of 12.8 months, and one patient exhibiting a strong T-cell response specific to the H3K27M mutation experienced a pseudoprogression, followed by a long-term complete remission lasting more than 31 months [80].

The Phase I ENACTING trial (NCT04749641) investigated a neoantigen peptide vaccine in 11 DIPG patients (7 post-surgery, 4 post-biopsy), achieving a median PFS of 11.4 months, a mOS of 15.4 months, a one-year survival rate of 66.7%, and one complete response. T cell responses validated specific T cell receptor clones recognizing the mutation [81]. The ongoing INTERCEPT H3 Phase I trial (NCT04808245) targets disseminated gliomas with the H3K27M mutation, expanding the vaccine’s potential applications [82].

### Isocitrate dehydrogenase R132H

The IDHR132H mutation, a common IDH1 mutation in glioma, alters the amino acid arginine for histidine at position 132, found in about 5% of primary GBMs despite its prevalence in low-grade gliomas and secondary GBMs [83]. However, the mutation alters the active site of the IDH, causing gliomagenesis, genetic instability, epigenetic hypermethylation, and differential synthesis of the oncometabolite D-2-hydroxyglutarate. The IDH1(R132H) peptide(p123-142: GWVKPIIIGHHAYGDQYRAT), a mutant IDH1 protein, has been shown to cause a mutation-specific CD4^+^ T-helper-1 (TH1) response in mice. This response, specific to mutations and restricted to MHC class II, effectively suppresses pre-existing IDH1(R132H)-expressing tumors. The peptide vaccine also inhibits the growth of IDH1(R132H) sarcomas without altering IDH1 wild-type enzymatic function. This suggests that the mutant epitope is processed internally and presented on MHC class II. This suggests that vaccination with the peptide vaccine could be a therapeutic approach [84].

A phase I study (NCT02454634) found that the IDH1-vac vaccine was well-tolerated and produced immune responses in 93.3% of patients with IDH1(R132H)-positive astrocytomas. The vaccine had an OS rate of 84% and a PFS rate of 63% after three years, with an 82% two-year PFS rate in patients who experienced immune reactions. The vaccine also identified mutation-specific T-helper cells in tumor-infiltrating T cells [85].

The safety, tolerability, and immunogenicity of the IDH1-vac vaccine in combination with the immune checkpoint inhibitor avelumab (AVE) in patients with IDH1R132H-mutant gliomas are being examined in the AMPLIFY-NEOVAC study (NCT03893903). This trial combines neoadjuvant and adjuvant immunotherapy in 48 evaluable patients with three treatment arms: IDH1-vac alone, combined with an immune checkpoint inhibitor, and an immune checkpoint inhibitor alone. Initial treatment lasts up to 43 weeks, followed by optional maintenance to enhance T-cell responses [86]. In the phase I clinical trial (NCT02193347), the peptide vaccine was tested in patients with grade 2 IDH1 R132H mutation glioma. The results showed a mutation-specific immunological response and a significant increase in IFN-γ + spot-forming splenocytes for the mutant IDH1 peptide [87]. Additionally, Researchers are conducting a clinical trial called ViCToRy (NCT05609994) to evaluate the safety and effectiveness of a PEPIDH1M vaccine combined with vorasidenib, a dual inhibitor of mutant IDH1 and IDH2 enzymes [88].

### Survivin

Survivin, a baculoviral IAP repeat-containing 5 (BIRC5) protein, regulates cell division and inhibits apoptosis by suppressing caspase activation. Overexpressed in numerous cancers, including GBM, it is detectable in 85% of tumor cells. Elevated survivin levels correlate with increased tumor proliferation, angiogenesis, therapy resistance, and poor prognosis. As a critical mediator of cytokinesis and cell cycle progression, survivin interacts with key signaling pathways, including p53, Wnt, hypoxia, TGF-β, and Notch. Its widespread expression and role in cancer biology position survivin as a promising immunotherapy target [89, 90]. SurVaxM, also known as SVN53-67/M57-KLH, is a synthetic peptide miming the human survivin protein sequence. Its modification of one amino acid (M57) enhances its binding to HLA-A*0201 molecules, and it is chemically bonded to KLH to enhance the immune response [91]. In the phase I clinical trial (NCT01250470) with nine rGBM patients expressing survivin, SurVaxM induced a cellular response in 6 and a local response in 3, showing high tolerability with no significant adverse events. The trial reported a median PFS of 17.6 weeks and a mOS of 88.6 weeks, with 7 patients surviving over 12 months [90]. A phase II clinical trial (NCT02455557) tested a combination of TMZ and the SurVaxM vaccine in 64 newly diagnosed glioblastoma patients. The treatment was well-tolerated, induced survivin-specific immune responses (CD8^+^ T cells and antibodies), and showed a median PFS of 11.4 months and OS of 25.9 months. It was effective in methylated and unmethylated patients, indicating broad clinical benefit. SurVaxM appears promising, with ongoing randomized trials assessing its efficacy [92]. Due to these positive outcomes, a phase II trial (NCT05163080) is ongoing to determine if combining SurVaxM with chemotherapy medication TMZ will increase survival rates for individuals with newly diagnosed GBM [93]. Phase II clinical research (NCT04013672) investigates the safety and effectiveness of combining SurVaxM with a PD-1 inhibitor (pembrolizumab) in treating rGBM patients [94]. Additionally, a pilot study (NCT04978727) evaluates the vaccine in children and young adults with specific brain tumors [95].

### **Wilm’s** tumor protein**-1**

Wilm’s tumor protein-1 (WT-1) is a protein involved in cell growth, apoptosis, differentiation, and organ development. It is overexpressed in hematological malignancies and solid tumors like GBM. Recent evidence suggests it’s involved in tumorigenesis as an oncogene [96]. A phase II clinical study on 21 patients with WT-1/HLA-A*2402-positive rGBM found that immunotherapy was well-tolerated, with a response rate of 9.5%, an illness control rate of 57.1%, a median PFS period of 20.0 weeks, and a 6-month PFS rate of 33.3% [97]. A small uncontrolled experiment confirmed the safety and clinical responsiveness of WT1 vaccination in rGBM patients, indicating the need for further research. A phase I trial in seven newly diagnosed GBM patients showed tolerability without severe hematological or neurological toxicities. However, 71.4% experienced grade 3 lymphocytopenia due to concurrent radiotherapy and TMZ therapy. Patients receiving WT-1 peptide vaccination in conjunction with TMZ showed a PFS ranging from 5.2 to 49.1 months [98]. A study found that the presence of WT1-235 IgG antibodies against the WT1 peptide in GBM patients receiving WT1 peptide vaccination was associated with long-term survival. These antibodies were detectable in 50.8% of cases, with Th1-type IgG1 and IgG3 subclasses present. The patient’s OS and PFS were positively correlated with antibody production. When combined with positive delayed-type hypersensitivity to the WT1-235 peptide, the WT1-235 IgG antibody is a superior prognostic marker for long-term OS [99]. Moreover, a phase I clinical trial evaluated a cocktail vaccine comprising WT1 HLA class I (CYTWNQMNL) and II (KRYFKLSHLQMHSRKH) peptides for safety and effectiveness in 14 HLA-A*24:02-positive patients with recurrent malignant gliomas. Most patients showed good tolerance, with mild grade I skin reactions at injection sites and stable disease six weeks after vaccination. No significant adverse effects were noted. A one-year survival rate of 36% was achieved, with a mOS of 24.7 weeks [100]. Trials are examining the safety and effectiveness of the WT-1 vaccine for GBM. In a phase I clinical trial (NCT02498665), the emulsion dose of DSP-7888 showed excellent tolerability and no dose-limiting toxicities. Intradermal injection led to a higher induction of WT1-specific cytotoxic lymphocytes, with a mOS of 180 days. WT1-specific CTL induction was observed in 66.7% of intradermally administered patients and 41.7% of subcutaneously administered patients [101]. In a separate Phase III clinical trial, registered as NCT03149003, DSP-7888 is being assessed in combination with bevacizumab for a cohort of 236 patients with rGBM [102]. WT2725 is a WT1-derived oligopeptide vaccine (RMFPNAPYL) designed to stimulate WT1-specific T-lymphocytes in HLA-A*0201^+^ and/or HLA-A*0206^+^ patients with WT1^+^ tumors. The phase I trial showed promising tolerability in advanced malignancies overexpressing WT1, such as glioblastoma, with no dose-limiting toxicities reported. Solid tumor patients had a median PFS of 2 months and a mOS of 13 months, with a notable immune-related response rate of 7.5%, particularly in the glioblastoma subgroup [103].

### TERT (Telomerase)

Telomerase, an anti-apoptotic enzyme, is upregulated in tumor cells and restores shortened telomeres after cell division, inhibiting replicative senescence. It consists of a catalytic component called human telomerase reverse transcriptase (hTERT) and a structural component called TERT. Mutations in the TERT gene are prevalent in central nervous system tumors, particularly gliomas, with the TERT promoter mutated in 80% of initial cases [104]. UCPVax is a therapeutic anti-cancer vaccine consisting of the telomerase-derived helper peptides UCP2 (TERT578–592:KSVWSKLQSIGIRQH) and UCP4 (TERT1041–1055: SLCYSILKAKNAGMS), designed to stimulate vigorous TH1 CD4 T cell responses in individuals diagnosed with cancer [105]. A Phase I/II clinical trial (NCT04280848) evaluating UCPVax combined with TMZ in GBM patients found that 97% developed an anti-TERT response after immunization, with 48% exhibiting an epitope spread response. The vaccine was well-tolerated, with a mOS of 17.9 months, a PFS of 8.9 months, and 26% of patients remaining alive two years post-diagnosis [106].

### Multipeptide vaccines

Several studies have evaluated the effectiveness of multi-peptide vaccines in reducing immune resistance and the recurrence of problems associated with single-peptide vaccinations, which incorporate several glioma-associated antigens (GAA).

A Phase II trial (NCT02078648) showed that patients with rGBM who received SL-701, a novel immunotherapy targeting EphA2, IL-13Rα2, and Survivin, in combination with synthetic peptides, poly-ICLC, and bevacizumab, achieved a 50% 12-month overall survival rate. Patients with varied T-cell responses did not show a direct correlation with survival. Patients surviving > 12 months had a 79% increase in SL-701-specific CD8 T-cells, a 36% decrease in CD4 T-cells, and a doubled CD8:CD4 ratio compared to those with shorter survival. They also had more (40% vs. 18%) CD57-expressing cytotoxic T-cells and a 20% lower CD57:CD107A ratio, indicating a replicating, non-terminally differentiated response. Immune response differences by week 8 may predict survival outcomes [107]. IMA950, a multi-peptide vaccine targeting TAAs like FABP7, BCAN, IGF2BP3, CSPG4, NRCAM, PTPRZ1, NLGN4X, TNC, c-met, and survivin, was tested with poly-ICLC in a phase I/II trial (NCT01920191) for newly diagnosed adult malignant astrocytoma patients. The vaccine was safe and well-tolerated, with four patients experiencing cerebral edema that resolved quickly. Initially, the vaccine-induced CD8 T-cell responses were limited to a single peptide for the first six patients, with no CD4 reactions observed. However, multi-peptide CD8 responses and sustained T helper 1 CD4 T-cell responses emerged after optimizing the vaccine formulation. In the overall cohort, 63.2% of patients exhibited CD8 T-cell responses to a single peptide, while 36.8% showed responses to multiple peptides. The mOS for GBM individuals was 19 months [108]. The clinical trial NCT03665545 investigates the combination of IMA950/Poly-ICLC with pembrolizumab for rGBM [109]. In a Phase 1/2 trial (NCT04116658), EO2401(EO), a peptide-based immunotherapy, was examined either alone or in combination with nivolumab (EN) or nivolumab plus bevacizumab (ENB) in 40 patients with GBM at first progression. In response to the EO, almost all patients exhibited strong CD8 T-cell responses, and most of them demonstrated verified cross-reactivity against tumor antigens. The treatments were well tolerated, and the EO’s adverse effects were limited to minor local responses. The 6-month os was 85% and 80% for EN and ENB, whereas the median PFS was 1.8 and 5.5 months, respectively. The objective response rates for EN and ENB were 36% and 10%, respectively. Bevacizumab improved PFS, and the trial is expanding to test low-dose bevacizumab for managing early neurological symptoms and prolonging exposure to EN [110]. P30-EPS, a peptide vaccine, is currently in phase 1b clinical trial (NCT05283109), recruiting participants to evaluate the immune profile in HLA-A*0201^+^ individuals with newly diagnosed, unmethylated, untreated grade IV malignant glioma [111]. Moreover, the Phase 1 clinical trial (NCT04842513) is recruiting participants for an immunomodulating multi-peptide vaccine using Pam3Cys-GDPKHPKSF (XS15) for patients with newly diagnosed MGMT-methylated glioblastoma who are HLA-A2-positive. This treatment is combined with radiation therapy and TMZ chemotherapy [112].

### Personalized peptide vaccine

A personalized peptide vaccine (PPV), derived from tumor-specific mutant antigens (TSMAs), stimulates the immune system against tumor cells. The vaccine, containing synthetic long peptides, can provoke a cytotoxic T-cell lymphocyte-mediated immune response.

In 2019, two phase I clinical trials assessed the safety and efficacy of a personalized neoantigen vaccine for GBM patients, focusing on its immune response and impact on patient outcomes and survival [113, 114]. The Phase I clinical trial (NCT02287428) explores the safety and feasibility of NeoVax, a personalized neoantigen vaccine combined with radiotherapy and pembrolizumab, in eight newly diagnosed GBM patients. Notably, the development of CD4 + and CD8 + T cell responses specific to the target neoantigen was limited to patients who had not received dexamethasone at the time of vaccination. About 20–30% of CD8 + and CD4 + T cell responses exhibited polyfunctionality, with half of these cells expressing at least one effector cytokine. This study showed that patients with T cells in the peripheral blood recorded approximately 7.6 months of PFS and 16.8 months of OS [114]. The GAPVAC-101 clinical trial (NCT02149225) involved 15 patients with newly diagnosed glioblastoma who received two sequential personalized therapeutic vaccines: APVAC1, targeting non-mutated antigens, and APVAC2, targeting neo-antigens. APVAC1 achieved 50% immunogenicity, eliciting primarily CD8 + T cell responses, while APVAC2 demonstrated 84.7% immunogenicity, predominantly inducing CD4 + T cell responses. This study found that the mOS was 29 months, and the median PFS was 14.2 months [113].

A phase III trial of PPV for HLA-A24^+^ rGBM patients, using four of 12 warehouse peptides selected based on pre-existing peptide-specific immunoglobulin G levels, showed no significant OS improvement compared to the placebo group. The control group had an OS of 8.0 months, while the experimental group had an OS of 8.4 months. The two groups had no statistical difference in median PFS [115]. The adverse effect of SART2-93 polypeptide selection was associated with clinical benefit. Patients who chose SART2–93 had an mOS of 6.6 months, which was much less than the 22.0 months of the control group. Increased numbers of CD4^+^CD45RA-activated T cells, decreased levels of CD11b^+^CD14^+^HLA-DR^low^ monocytes, and intermediate levels of CCL2, VEGF, IL-6, IL-17, and haptoglobin are biomarkers of improved OS in PPV patients.

The Phase I clinical trial (NCT 03422094) combined therapy with nivolumab and ipilimumab in MGMT-unmethylated GBM patients using the NeoVax vaccine. The study found a significant increase in IFNg-producing T cells in PBMCs from subjects 1–3 after receiving NeoVax neoantigens, indicating a robust immune response to the vaccine. Furthermore, the tumor biopsy of Subject 3 revealed infiltrating, clonally increased T cells after NeoVax therapy, suggesting a favorable immunological reaction to the vaccine [116]. Moreover, a clinical trial (NCT03223103) is currently underway to evaluate the safety, tolerability, and immunogenicity of a personalized vaccine based on mutation-derived tumor antigens (MTA) in patients recently diagnosed with glioblastoma and undergoing continuous Tumor Treating Fields (TTFields) treatment [117].

### Adjuvants

Vaccine adjuvants are immunostimulatory elements in vaccine technology that stimulate the immune system, increasing the immunogenicity and effectiveness of the vaccine. Given the limited capacity of cancer peptide vaccines to elicit an immune response, adjuvants are likely to enhance the stimulation of effective immune responses targeting specific antigens [118]. Once tumor antigens are injected in vivo, the adjuvant usually introduces damage-associated molecular patterns (DAMPs) or pathogen-associated molecular patterns (PAMPs) to stimulate the innate immune system. As a result of activation, co-stimulatory molecules are released, cytokines are released, and innate immune cells are recruited to mount adaptive immune responses [119, 120]. In GBM vaccination studies, the adjuvants poly-ICLC, tetanus toxoid, montanide, imiquimod, GM-CSF, and CpG-ODN are the most effective and employed. Polyinosinic–polycytidylic acid (poly (I: C)), or Hiltonol, known as a Toll-like receptor 3 (TLR3) agonist, efficiently elicits strong cytotoxic CD8^+^ T cell responses against tumors. This synthetic double-stranded RNA (poly (I: C)) can be stabilized with poly-L-lysine to create ploy-ICLC, an RNase-resistant analog. This substance can activate particular T cell populations, which direct the immune system to attack and eliminate tumor cells. In GBM vaccination clinical studies, poly-ICLC is often employed (NCT03665545, NCT05557240, NCT03223103, NCT02510950, NCT02754362, NCT02078648, NCT03422094).

Montanide is a clinical-grade Incomplete Freund’s Adjuvant, a water-in-oil emulsion that creates an antigen depot and gradually releases antigens to increase antigen bioavailability. Montanide ISA 51 and Montanide ISA 720 are two formulations of Montanide used as adjuvants in human vaccination studies. Mannide monooleate and mineral oil are used to emulsify Montanide ISA 51, whereas non-mineral oil and mannide monooleate are used in the case of Montanide ISA 720. The production of CTL and serum antibodies is increased by Montanide ISA 720 and ISA 51 adjuvants, according to the results of preclinical and clinical trials. Nevertheless, it can eliminate long-lasting CD8 + T-cell immunity and induce T-cell sequestration at the immunization site [121]. Montanide ISA 51 was utilized in GBM vaccine clinical trials (NCT04978727, NCT04013672, NCT02455557, NCT04842513, NCT02754362, NCT02864368).

The administration of tetanus toxoid (TT) before vaccination has demonstrated encouraging outcomes in augmenting the effectiveness of DC vaccines for treating glioblastoma. Research has indicated that TT pre-conditioning before DC vaccination can enhance the homing of lymph nodes, DC migration, and overall survival rates in individuals with GBM [122, 123]. An efficient immune response against GBM has been facilitated by the combination of TT pre-conditioning with DCs loaded with autologous tumor lysate, which has vigorously activated CD8^+^ T-cells, formed memory T-cells, and generated helper T-cells [122]. Thus, clinical trials (NCT 02864368) and (NCT02193347) investigated the tetanus-diphtheria toxoid concerning peptide vaccines against GBM.

Granulocyte-macrophage colony-stimulating factor, or GM-CSF, is a vital cytokine growth factor that is well-known for its capacity to stimulate and increase the generation of monocytes, eosinophils, and neutrophils, all of which are essential components of the immune response. Studies on vaccines have demonstrated that the adjuvant causes neutrophil, NK cell, and macrophage activation as well as DC maturation and recruitment [118]. To date, GM-CSF has been utilized in various clinical trials (NCT00643097, NCT00643097, NCT01498328, NCT01480479, NCT01222221) in combination with peptide vaccines and drugs.

The intrinsic release of double-stranded DNA (dsDNA) from cancer cells triggers the activation of the STING pathway, a vital biological mechanism that serves as a sensor for the existence of core cytosolic dsDNA in the cellular environment. Its activation inhibits the development of tumor cells from the outset by encouraging the infiltration of immune cells, such as NK and T cells, and increasing the production of proinflammatory cytokines, such as chemokines and type I interferons (118). Another possible adjuvant for activating tumor vaccines is STING agonists. Compared to GM-CSF alone, its use dramatically increases T-cell infiltration and dose-dependently reduces tumor growth [124]. The importance of IFN-stimulated genes in the clinical outcome of GBM patients is demonstrated by the discovery of a three-gene risk model combining STAT3, STAT2, and SOCS3 as an independent predictor in the context of GBM [125].

Imiquimod, a synthetic imidazoquinoline, stimulates TLR7/8 to activate the immune system and improve the body’s capacity to identify and tackle tumor cells [118]. Studies reveal that imiquimod, independently of TLR/MYD88 signaling, can directly suppress Hedgehog (HH) signaling by adversely modifying GLI activity in basal cell carcinoma and medulloblastoma cells [126]. Single-stranded RNA activates TLRs 7 and 8, which in turn upregulate costimulatory molecules (CD80/86 and CD40), boost cytokine production (IFN-alpha, TNF-alpha, and IL-12), and improve the migration of DCs from lymph nodes [118]. A clinical trial I (NCT04808245) tested a peptide Vaccine trial for the treatment of H3-mutated gliomas of imiquimod as a vaccine adjuvant.

Synthetic compounds known as CpG oligonucleotides (CpG ODN) comprise unmethylated cytosine-guanine dinucleotide patterns. These molecules stimulate TLR9 and initiate innate immune responses characterized by releasing Th1 and proinflammatory cytokines [127]. Following the addition of a peptide vaccine from hTERT to CpG oligodeoxynucleotides (CpG-ODN), a phase I clinical study in GBM patients demonstrated a significant increase in T cell chemoattraction [128].

A unique water-soluble synthetic derivative of Pam3Cys, XS15, is being developed as a possible adjuvant for peptide vaccination and has demonstrated encouraging results in boosting immune responses. Studies reveal that when XS15 is mixed with peptides in a water-in-oil emulsion, it stimulates peptide-specific CD8^+^ T and NK cells, activates immune cells, and elicits functional immunological responses in humans [129]. In addition, interleukin-15 (IL-15) is another effective adjuvant that may be used alone or in combination with other adjuvants to enhance immune responses to vaccines [130]. These results demonstrate the ability of XS15 and IL-15 to boost immune cell activation and stimulate robust immunological responses, hence increasing vaccination efficiency. As such, they are promising candidates for further vaccine development and the creation of customized tumor peptide vaccines.

### Developing effective vaccines for GBM is particularly challenging due to several factors (Fig. 3)

**Fig. 3:**
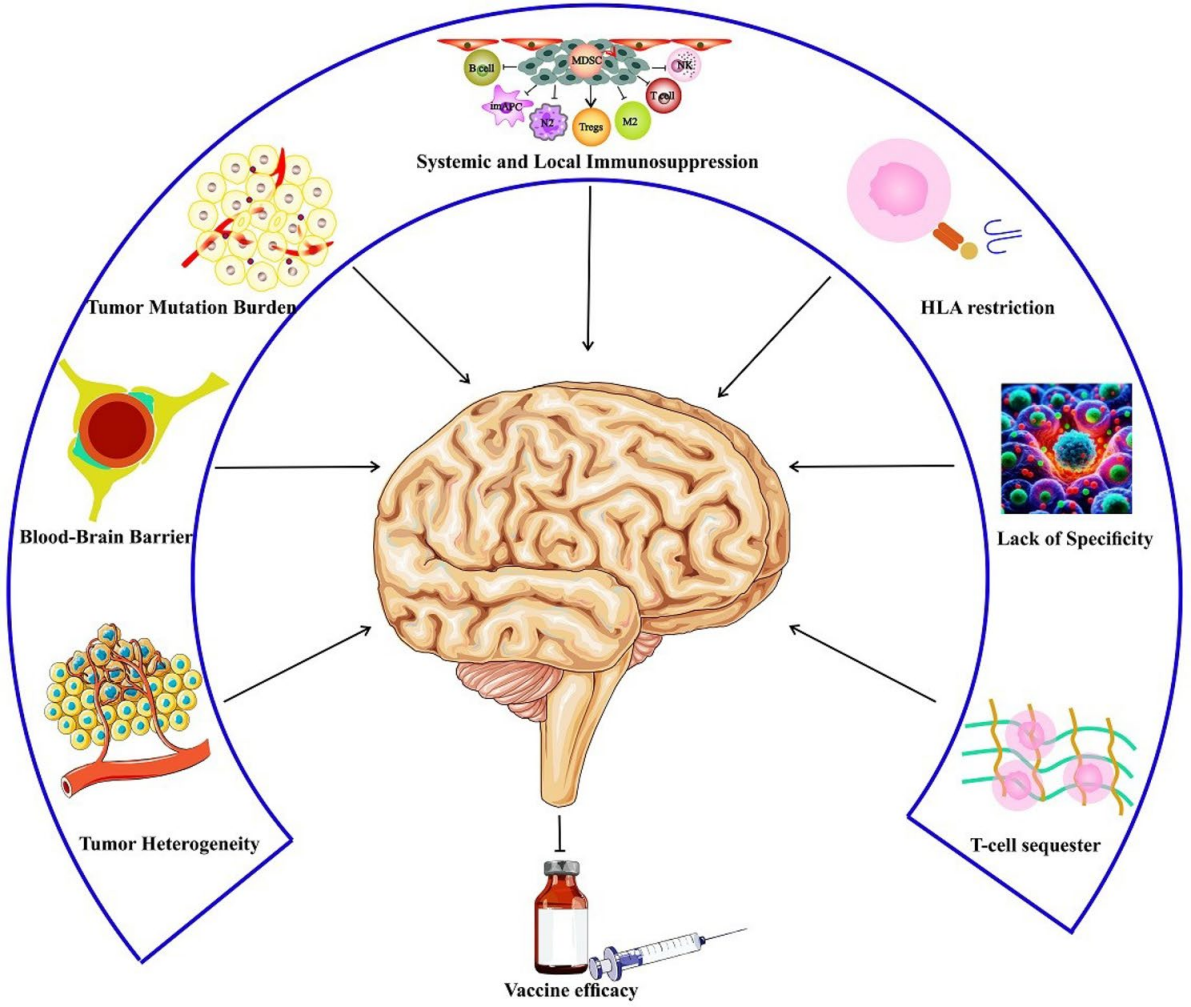
Factors affecting vaccine efficacy in glioblastoma multiforme

#### Tumor heterogeneity

Tumor heterogeneity significantly impacts vaccine development in GBM by influencing the selection of suitable tumor antigens and patient-specific immunotherapies. The heterogeneity of these tumors poses a challenge for immunotherapy strategies, including vaccines. A summary of the impact of tumor heterogeneity on vaccine efficacy in glioma:

A. **Vaccine Development**: Due to the heterogeneous genetic composition of glioma cells, a vaccine targeting a specific tumor antigen may not work on all tumor cells. This is because distinct cells distinct cells harbor unique antigens.
B. **Clinical Trials**: Phase III clinical studies have demonstrated minimal effectiveness for vaccines such as the anti-EGFRvIII ridopepimut (CDX-110). This is partially caused by the heterogeneity of glioblastoma, which hinders the substantial effect of a single antigen vaccination [131]. Also, Diffuse midline glioma is known to carry the H3.3K27M mutation, which is not well processed by HLA-A restricted CD8^+^ T cells and hence not suited for cancer immunotherapy [132].
C. **Immunotherapy Strategies**: The goal is to create vaccines against neoantigens and combine them with immunomodulators to overcome the challenges presented by tumor heterogeneity. By focusing on the distinct antigens found in each patient’s tumor, these tactics aim to enhance individualized therapy and maybe increase efficacy [131].
D. **Cell-based Vaccines**: Clinical trials have shown promise for cell-based vaccines using dendritic and tumor cells. Patients with GBM have been shown to have somewhat improved PFS and overall recovery. However, developing potent antigens to stimulate immunological responses is still required [133].
E. **Combination Therapies**: Increasing the effectiveness of a vaccine may involve combining several antigens, such as TAAs, neoantigens, and pathogen-derived antigens, as well as altering the vaccine design or delivery system [55].

### Systemic and local immunosuppression

By erecting obstacles to effective immunotherapy, systemic and local immunosuppression significantly influences the development of GBM vaccines. The intricate interactions between different immune cells within the tumor microenvironment are responsible for local immunosuppression in GBM. The activities of antigen-presenting, helper, and effector immune cells are disrupted, and resulting in a reduction in T lymphocytes and an increase in the proliferation of immunosuppressive cells, such as macrophages, microglia, MDSCs, and Tregs [134].

Immunosuppressive subsets in the blood and alterations in circulating immune cells are two indicators of systemic immunosuppression. One such metric investigated for its predictive value in GBM patients is the neutrophil-to-lymphocyte ratio (NLR) [134].

Studies have demonstrated that GBM causes systemic immunosuppression characterized by decreases in peripheral T-cell counts, thymic and splenic involutions, and reduced CD4 T-cell counts [135]. Immunotherapeutic techniques, such as therapeutic vaccines, are hampered by this immunosuppression because it leads to T-cell changes, lymphopenia, and an increase in MDSCs. To further prevent vaccine-induced immune responses, the immunosuppressive environment in GBM is exacerbated by high concentrations of Treg cells and serum factors that limit T cell proliferation and activity [136].

### Tumor mutation burden (TMB)

Tumor-specific antigens and tumor neoantigens are distinct molecular markers particular to cancer cells. They enable the immune system to differentiate them from normal cells and initiate an immunological response; however, GBM has significantly lower amounts of both antigens. TMB is a reliable indicator of the effectiveness of immunotherapy in non-CNS cancers [137]. Individuals with low TMB with rGBM who undergo immunotherapy treatments like immune checkpoint blockade or PVSRIPO show longer survival. This might be attributed to an improved immune response via immune editing or neoantigen depletion. Immunotherapy may help treat de novo hypermutated glioma, a rare condition primarily caused by a mutation in the POLE gene or a defect in the mismatch repair (MMR) gene. Hypermutation, often observed in recurrent stages after TMZ administration, is subclonal with diminished immunogenicity, indicating a lack of significant correlation with immunotherapy benefits [138].

### Blood-brain barrier (BBB)

The BBB presents a significant obstacle to developing a GBM vaccination because it can limit the entrance of immune cells and macromolecules into the central nervous system (CNS). In rGBM, multiple mechanisms cause the BBB to change significantly, complicating therapeutic approaches: (A). Increased Permeability (Blood-Tumor Barrier, BTB): The BBB is often compromised in rGBM, resulting in a more permeable BTB inside tumor areas. This change is the result of several factors. Vascular endothelial growth factor (VEGF) overexpression causes angiogenesis, which leads to aberrant, leaky blood vessels with fenestrated endothelium and compromised tight junction integrity. Changes in tight junctions, such as decreased expression of proteins like occludin and claudin-5, impair endothelial cell connections and increase paracellular permeability. Furthermore, glioma cell invasion compromises the blood-brain barrier as tumor cells penetrate perivascular spaces, break down the extracellular matrix, and interfere with astrocyte-endothelial interactions. (B). Heterogeneity Across Tumor Regions: The BTB exhibits differences in different tumor areas. While the invasive periphery keeps the BBB largely intact, the tumor core usually shows a highly disrupted BTB, which limits the ability of drugs to reach infiltrating tumor cells. Recurrence-specific changes further complicate this landscape, as prior treatments (radiation, TMZ) exacerbate BBB/BTB damage, increasing permeability and inducing fibrosis or necrosis, impeding effective drug distribution. (C). Efflux Transporter Overexpression: rGBM and endothelial cells frequently overexpress efflux transporters, including P-glycoprotein (P-gp) and breast cancer resistance protein (BCRP), despite elevated BTB permeability. These transporters decrease the effectiveness of treatment, actively removing medications from the brain. (D). Inflammation and Immune Cell Infiltration: the inflammatory microenvironment created by rGBM is further compromised by cytokines such as TNF-α and IL-6, which break tight junctions and increase BBB permeability. The BBB is further disrupted by immune cell infiltration (such as macrophages and T-cells), but this is insufficient to provide reliable drug delivery [139–141]. Ultrasound-assisted microbubble-mediated BBB opening is one promising strategy to improve drug delivery across the BBB and BTB in rGBM. Other strategies aim to increase permeability or temporarily circumvent these barriers. Gas-filled microbubbles (1–10 μm), usually lipid- or protein-shelled and FDA-approved for imaging (e.g., Definity, Optison), are injected intravenously. Low-intensity focused ultrasound (FUS) waves (0.5–1.5 MHz) are then applied transcranially to a specific brain region. The microbubbles oscillate or collapse due to stable or inertial cavitation induced by ultrasound, mechanically stressing endothelial cells and temporarily loosening tight junctions to enhance paracellular transport. This procedure enhances drug delivery to the tumor site by increasing BBB permeability for 4–24 h, depending on the duration, intensity, and dose of microbubbles [142–144]. Preclinical studies in rGBM mouse models demonstrate that focused ultrasound (FUS) enhances the delivery of temozolomide and bevacizumab, resulting in significantly improved survival and reduced tumor growth. Clinically, early-phase trials (e.g., NCT03616860, NCT04440358) are assessing FUS with microbubbles in rGBM patients, confirming safety and feasibility. A 2023 study using the NaviFUS device reported successful BBB opening, evidenced by increased gadolinium contrast uptake, indicating enhanced permeability [145, 146]. These BBB and BTB alterations limit the effectiveness of traditional immunotherapies by preventing immune cells from entering the tumor microenvironment. This inhibits the activation of the adaptive immune response and decreases the ability of autoreactive lymphocytes to enter the tumor [147].

### Lack of specificity

In many cases, tumor antigens are not generated by mutations but rather by aberrant or overexpression of normal proteins found in other tissues. This occurrence highlights the intricate relationships between protein regulation and genetic alterations during cancer development. In this situation, antigen targeting may lead to autoimmunity, which has unwanted consequences, including brain inflammation. The development of techniques based on peptide vaccines is limited in GBM due to the lack of specific epitopes. The high expression levels of epitopes and the general lack of specificity found in GBM significantly impact the development of peptide vaccination methods [148].

### HLA restriction

One of the main obstacles to the therapeutic use of TAA is the phenomenon known as HLA restriction, defined as more than 25,000 variations in the human population [149]. The range of clinical studies that examine the effectiveness of GBM TAAs is primarily limited to people who bear the HLA-A2, HLA-A24:02, or HLA-A 03 allotype, perhaps resulting in reduced efficacy for patients with varying HLA haplotypes [90, 100, 108, 112, 150]. This restriction emphasizes the need for further study and development to increase the compatibility of TAA-based treatments across a range of HLA profiles. Moreover, apart from the currently recognized TAAs, other unidentified TAA possibilities need investigation, including transposable elements and alternative splicing.

### T-cell sequester

T-cell sequestration in GBM is caused by the tumor-induced loss of S1P1 from T-cell surfaces, both CD4^+^ and CD8^+^. As in GBM mice and treatment-naïve patients, this results in the accumulation of T cells in the bone marrow rather than in the peripheral blood or circulating secondary lymphoid organs. As a result, T-cell infiltration into the GBM tumor site is reduced [151]. Reversing T cell sequestration through surface stabilization of S1P1 has demonstrated potential in approving T cell-activating treatments in GBM murine models. Moreover, it has proven possible to reverse T cell sequestration in the bone marrow of GBM-bearing animals, increase T cell infiltration into tumors, and raise overall survival rates by repurposing medications like paroxetine combined with biomimetic nanoparticles [152]. T cell dysfunction results from this sequestration and exhaustion, which reduces the ability of T cells to kill tumor cells through cytotoxicity. T-cell dysfunction in GBM is exacerbated by several immunological checkpoints, such as PD-1, TIM-3, and LAG-3, which reduce cytokine release and decrease effector activities [153].

## Conclusion

The aggressive nature of GBM renders it a severe disease, with few viable treatment options and inadequate symptom management. Consequently, there is an urgent need for safer and more targeted antitumor interventions to address these limitations. Immunotherapy is gaining recognition as a promising treatment for malignant brain tumors, a complex and delicate brain organ that presents significant challenges in effective therapeutic interventions. A vaccination developed specifically for GBM tumors could represent a valuable adjunctive treatment option, enhancing the effectiveness of the current standard-of-care regimen. Personalized multi-peptide vaccines, which combine neoantigens and tumor-associated antigens, have shown enhanced tumor regression outcomes and potent anti-tumor immune responses compared to traditional single-peptide vaccination strategies. Epigenetic mechanisms in gliomagenesis suggest that there are limited neoantigens for immunotherapy targeting GBM, which could be addressed by expanding and diversifying vaccineantigens, potentially enhancing therapeutic intervention. The methodology demonstrated a satisfactory safety profile and a long-lasting memory response in CD8^+^ T cells, while also effectively targeting predicted neoepitopes from CD4^+^ T cells. Improving vaccine design, particularly through the careful selection of antigens and adjuvants, while focusing on the role of CD4^+^ T cells in tumor-targeted vaccines, alongside the incorporation of combination immunotherapy, will be crucial strategies for enhancing the efficacy of vaccines aimed at treating GBM.

## Data Availability

No datasets were generated or analysed during the current study.

## Abbreviations

GBM: Glioblastoma multiforme
TSAs: Tumor-specific antigens
TAAs: Tumor-associated antigens
MDSCs: Myeloid-derived suppressor cells
GAMs: Glioma-associated macrophages/Microglia
Tregs: Regulatory T Cells
ICIs: Immune checkpoint inhibitors
CTLA-4: Cytotoxic T lymphocyte-associated protein
PD-1: Programmed cell death protein
LAG-3: Lymphocyte-activation gene 3
IDO1: Indoleamine 2,3-dioxygenase 1
Tim-3: T-cell immunoglobulin or Mucin-domain containing-3
TIGIT: T cell immunoreceptor with Ig and ITIM domains
APCs: Antigen-presenting cells
EGFRvIII: Epidermal growth factor receptor variant type III
IDHR132H: Isocitrate Dehydrogenase R132H
TERT: Telomerase reverse transcriptase
WT-1: Wilm’s tumor protein-1
PPV: Personalized peptide vaccine
TSMAs: Tumor-specific mutant antigens
CTLs: Cytotoxic T lymphocytes
HTLs: helper T lymphocytes
